# The contribution of ferroptosis to the epithelial-mesenchymal transition phenotype in models of age-related macular degeneration

**DOI:** 10.3389/fcell.2026.1718715

**Published:** 2026-02-13

**Authors:** Xingyu Yang, Weichen Xu, Hang Xie, Xiaoyu Guo, Feiyan Zhu, Yiqiao Xing, Changzheng Chen

**Affiliations:** 1 Eye Center, Renmin Hospital of Wuhan University, Wuhan, China; 2 Eye Institute of Wuhan University, Wuhan, China

**Keywords:** age-related macular degeneration (AMD), cellular senescence, epithelial-mesenchymal transition (EMT), ferroptosis, oxidative stress, retinal pigment epithelium (RPE)

## Abstract

**Purpose:**

Age-related macular degeneration (AMD) involves dysfunction of the retinal pigment epithelium (RPE), where cellular senescence and epithelial-mesenchymal transition (EMT) are key pathological features. The upstream mechanisms linking these processes are not fully understood. This study investigates the potential role of ferroptosis in contributing to senescence-associated EMT in RPE cells.

**Methods:**

We utilized an aging mouse model and two cellular models in ARPE-19 cells, induced by D-galactose (D-gal) and low-dose sodium iodate (SI), respectively. Ferroptosis, EMT, and oxidative stress markers were evaluated via immunofluorescence, flow cytometry, and Western blotting. The specific ferroptosis inhibitor Ferrostatin-1 was used to assess the involvement of ferroptosis.

**Results:**

Aged mouse RPE/choroid complexes and stressed ARPE-19 cells exhibited features of EMT along with increased ferroptosis hallmarks, including lipid peroxidation and iron accumulation. A downregulation of the xCT/GPX4 anti-ferroptotic axis was observed. Pretreatment with Fer-1 alleviated ferroptosis by reducing iron levels and lipid peroxidation, and restored xCT/GPX4 expression. Furthermore, Fer-1 attenuated the EMT phenotype, as evidenced by the restoration of epithelial markers and reduction of mesenchymal markers (Vimentin, α-SMA) in both D-gal and SI models.

**Conclusion:**

Our findings suggest that ferroptosis may contribute to linking RPE senescence with EMT, potentially via oxidative stress pathways. The combined targeting of both senescence and ferroptosis could therefore represent a potential therapeutic strategy for addressing RPE dysfunction and AMD progression.

## 1 Introduction

Age-related macular degeneration (AMD) is the leading cause of irreversible vision loss in the elderly worldwide, and its prevalence rises markedly with advancing age (Guymer and Campbell, 2023; Girgis and Lee, 2023). Retinal pigment epithelium (RPE) cells play an important role in protecting photoreceptor function and maintaining retinal homeostasis, with its structural integrity and functional competence being essential for sustained visual performance (Mitchell et al., 2018; Jabbehdari and Handa, 2021). Therefore, elucidating the molecular mechanisms driving age-associated degenerative changes in RPE cells is essential for the exploration of effective therapeutic interventions.

Epithelial-mesenchymal transition (EMT) is a key pathological remodeling process in RPE cells, contributing to outer blood-retinal barrier disruption and subretinal fibrosis in advanced AMD (Yao et al., 2022; Liu et al., 2023; Zhou et al., 2020). In EMT process, RPE cells lose their characteristic cell polarity and intercellular junctions, and gain enhanced migratory capacities (Shen et al., 2023; Yang et al., 2024; Higashijima et al., 2022). Elevated levels of EMT biomarkers have been detected from the serum of AMD patients (Liukkonen et al., 2022). While pathways like TGF-β are known to induce EMT (Jiang et al., 2025; Jang et al., 2023; Wang et al., 2025a), the upstream triggers within the context of aging and chronic oxidative stress, as hallmarks of the AMD microenvironment, remain less defined.

Cellular senescence and oxidative injury are pivotal drivers of AMD pathogenesis (Xu et al., 2023; Terao et al., 2024; Terao et al., 2022). Senescent RPE cells exhibit altered function and a pro-inflammatory secretory phenotype, while chronic oxidative stress directly damages cellular components (Terao et al., 2022; Lei et al., 2023; Lee et al., 2021). Both processes can promote a tissue microenvironment conducive to pathological remodeling, including EMT (Ortiz-Montero et al., 2017; Qin et al., 2024).

Ferroptosis, an iron-dependent form of regulated cell death driven by lipid peroxidation, has emerged as a significant mechanism in aging, neurodegeneration, and fibrotic diseases (Chen et al., 2021; Zhang et al., 2024). Central to this process is lipid peroxidation and suppression of glutathione peroxidase 4 (GPX4) activity, a mechanism intrinsically linked to oxidative stress injury (Liu et al., 2024; Zhu and Yu, 2024).Its core biochemical process is intrinsically linked to oxidative stress and glutathione metabolism, placing it at the intersection of key AMD pathogenic factors (Gupta et al., 2023; Xiang et al., 2024; Tang et al., 2021). Notably, emerging evidence suggests crosstalk between ferroptosis and cellular phenotypic plasticity, including EMT programs in other contexts (Han et al., 2025; Ebrahimi et al., 2022; Zhang et al., 2024; Schwab et al., 2024). This raises a compelling translational question: could targeting ferroptosis mitigate the detrimental phenotypic shifts in RPE cells under AMD-relevant stressors?

To explore this, we began by observing elevated levels of ferrous iron (Fe^2+^), malondialdehyde (MDA), and EMT markers in the RPE/choroid complex of aged mice, a model that recapitulates early pathological changes relevant to the onset of AMD. To mechanistically investigate these observed changes, we then established two complementary cellular models of AMD-relevant stress: D-galactose (D-gal) to induce stress-induced premature senescence, and low-dose sodium iodate (SI) to model oxidative injury. We found that both treatments could elicit an EMT-like phenotype in RPE cells. Building on this, we then used the specific inhibitor Ferrostatin-1 (Fer-1) to test whether blocking this pathway could reduce the EMT features induced by D-gal and SI. This study, therefore, aims to evaluate the contribution of ferroptosis to stress-associated EMT in RPE cells and explore its potential as a therapeutic target for AMD intervention.

## 2 Materials and methods

### 2.1 Animals

Male C57BL/6J mice (8-week-old, young; and 18-month-old, aged) were purchased from the Experimental Animal Center of Wuhan University. All animal procedures strictly adhered to the ARVO Statement for the Use of Animals in Ophthalmic and Vision Research and were approved by the Institutional Animal Care and Use Committee of Wuhan University. Following euthanasia by cervical dislocation, eyeballs were promptly enucleated and transferred to ice-cold phosphate-buffered saline (PBS). The anterior segment and the sclera were sequentially removed by gentle mechanical separation to obtain the RPE/choroid complex. All subsequent dissections were performed on a chilled stage or over ice using a stereomicroscope and fine ophthalmic instruments.

### 2.2 Cell culture

The adult human retinal pigment epithelium cell line ARPE-19 (Pricella, China) was cultured in Dulbecco’s Modified Eagle Medium/Nutrient Mixture F-12 (DMEM/F-12, Gibco) supplemented with 10% fetal bovine serum (FBS, Gibco) and 1% penicillin-streptomycin (Gibco). To promote a differentiated, cover slips and culture plates were pre-coated with Laminin (10 μg/mL, Biolamina) overnight at 4 °C (Bernd et al., 2024). Cells were then seeded at a high density of 1.6 × 10^5^ cells per cm^2^ in standard tissue culture plates (Corning). For experiments requiring protein or cell collection (Western blot, flow cytometry, ELISA), cells were cultured in 6-well plates. For microscopic analyses (immunofluorescence, SA-β-galactosidase staining), cells were grown on cover slips placed or without cover slips in 24-well plates. For the CCK-8 assay, cells were seeded into 96-well plates.

Once reaching confluence, the culture medium was replaced with differentiation medium containing 10 mM nicotinamide (MCE) to induce polarization and functional maturation (Hazim et al., 2019). Cells were maintained in this post-confluent, differentiation-promoting state for a minimum of 2 weeks before all experiments, with the medium changed every 2–3 days. All experiments were performed using cells between passages 3 and 6. Cells were maintained at 37 °C in a humidified incubator with 5% CO_2_.

For the senescence model, cells were treated with D-galactose (200 mM for 72 h). For the oxidative injury model, cells were treated with sodium iodate (2 mM for 48 h). To inhibit ferroptosis, cells were pre-treated with 20 μM Ferrostatin-1 (Fer-1, MedChemExpress) for 2 h before and during D-gal or SI exposure (Chen et al., 2023). An equal volume of phosphate-buffered saline (PBS) or dimethyl sulfoxide (DMSO, final concentration <0.1%) served as vehicle control for D-gal/SI and Fer-1.

### 2.3 Microscopy and immunofluorescence

Bright-field images for morphological assessment were acquired using an Olympus microscope equipped with a 10× objective lens.

For immunofluorescence, cells on coverslips were fixed with 4% paraformaldehyde (15 min, room temperature), permeabilized with 0.1% Triton X-100 (10 min), and blocked with 5% BSA (1 h). Samples were then incubated with primary antibodies diluted in 5% BSA overnight at 4 °C. After washing, they were incubated with species-appropriate fluorophore-conjugated secondary antibodies (Antgene, ANT024, ANT030, 1:500) for 1 h at room temperature in the dark. Nuclei were counterstained with DAPI (Beyotime, P0131). Primary antibodies and dilutions used were: Vimentin (1:500, ZenBio, #R22775) and α-Smooth Muscle Actin (α-SMA, 1:200, Abcam, ab5694). Immunofluorescence image acquisition was performed using an Olympus IX73P1F inverted fluorescence microscope. For direct comparison, all images within a given experiment were captured using the same exposure time and imaging parameters.

For quantitative comparison within an experiment, all images for a given marker were acquired using the same objective lens and identical exposure/gain settings. Quantification of mean fluorescence intensity (MFI) was performed on images captured with a 20× objective from at least five random fields per experimental condition. Additional high-resolution representative images were obtained using a ×100 oil immersion objective.

Image quantification was performed using ImageJ software (NIH). The region of interest (ROI) was outlined for each cell or field, and the background-subtracted MFI was calculated. For each independent experiment, the MFI values were normalized to the average MFI of the control group (set as 1).

### 2.4 Cell death quantification and cell viability measurement

Cell viability was assessed using the Cell Counting Kit-8 (CCK-8, Beyotime, C0037). Briefly, after the indicated treatments, the culture medium was replaced with fresh medium containing 10% (v/v) CCK-8 reagent. Cells were then incubated at 37 °C for 2 h. The absorbance at 450 nm was measured using a microplate reader. For each experimental condition within an individual experiment, the average absorbance of multiple technical replicates was calculated and used as the representative value. This value was then normalized to the average of the control group (set as 1) to generate the relative cell viability. The final results presented are from three independent experiments performed on different days using distinct cell passages.

### 2.5 Measurement of intracellular reactive oxygen species (ROS) and lipid peroxidation

Intracellular ROS levels were measured using the fluorescent probe DCFH-DA (Beyotime, S0033S). After treatments, cells were incubated with 10 μM DCFH-DA for 30 min at 37 °C in the dark, washed, and the fluorescence intensity was immediately measured by flow cytometry (Beckman CytoFLEX).

To specifically assess lipid peroxidation, a hallmark of ferroptosis, cells were loaded with 10 μM C11-BODIPY 581/591 (Thermo Fisher, D3861) for 30 min at 37 °C. After washing, the fluorescence of the oxidized forms was analyzed by flow cytometry. The flow cytometry gating strategy was as follows: cells were first gated on forward scatter (FSC-A) vs. side scatter (SSC-A) to exclude debris, followed by gating on FSC-A vs. FSC-H to select single cells. For each independent experiment, the fluorescence-positive gate was first established based on unstained blank cells, such that ≥99.5% of this population was designated as negative. This identical gate was then applied uniformly to all stained samples within that experiment. Both the geometric mean fluorescence intensity (MFI) and the percentage of positive cells were recorded for analysis. To account for inter-experimental variation, the MFI values were normalized to the control group, the mean MFI of which was set as 1.

### 2.6 Senescence-associated β-galactosidase (SA-β-gal) staining

Cellular senescence was detected using a SA-β-Gal staining kit (Beyotime, C0602) following the manufacturer’s instructions. After treatment, cells were fixed and incubated with the staining working solution at 37 °C for 16 h. SA-β-Gal-positive cells, identified by the development of blue cytoplasmic staining, were visualized and imaged using the previously described Olympus IX73P1F inverted microscope equipped with a 10× objective. To quantify the senescence rate, the percentage of SA-β-Gal-positive cells was determined by counting cells from at least five random fields per condition using ImageJ software.

### 2.7 Western blot analysis

Total protein was extracted from cells or RPE/choroid complexes using RIPA lysis buffer containing protease and phosphatase inhibitors. Protein concentration was determined by the BCA assay. Equal amounts of protein (20–30 μg) were separated by SDS-PAGE and transferred to PVDF membranes. Membranes were blocked with 5% non-fat milk and incubated with primary antibodies overnight at 4 °C, followed by HRP-conjugated secondary antibodies for 1 h. Signals were developed using an ECL kit and captured with a chemiluminescence imaging system. The intensity of each target protein band was normalized to that of its corresponding β-actin loading control. For statistical analysis across independent experiments, the normalized values of the experimental groups were further calibrated against the mean value of the control group within the same experiment, which was set as 1.

The following antibodies were used: β-catenin (Proteintech, 51067-2-AP), Vimentin (zenbio, #R22775), E-cadherin (zenbio, #R22490), ZO-1 (Affinity, AB_2837631), N-cadherin (Abmart, T55015), GPX4 (Proteintech, 67763-1-Ig), β-actin (Abclonal, AC026), xCT (Proteintech, 26864-1-AP), p21 (Abmart, TA5484F), p16INK4a (Abmart, T55543F),HRP-conjugated Goat Anti-Mouse IgG (H + L) (Proteintech, SA00001-1), HRP-conjugated Goat Anti-Rabbit IgG (H + L) (Proteintech, SA00001-2).

### 2.8 Periodic Acid-Schiff (PAS) staining

Retinal morphology and the integrity of the RPE basement membrane were evaluated using Periodic Acid-Schiff (PAS) staining on paraffin-embedded sections (4 µm). After deparaffinization in xylene and hydration through a graded ethanol series, sections were oxidized in 0.5% periodic acid (Sigma-Aldrich) for 10 min at room temperature. Following thorough rinsing in distilled water, sections were incubated in Schiff’s reagent (Sigma-Aldrich) for 20 min in the dark. Slides were then washed in lukewarm running water for 5 min to develop the characteristic magenta color. Cell nuclei were lightly counterstained with Mayer’s hematoxylin for 30 s. After a final dehydration through graded alcohols, clearing in xylene, and mounting with a permanent mounting medium, slides were imaged under a bright-field microscope (Olympus). Representative images were acquired at both 20× and 100× magnification. The staining highlights glycoproteins, basement membranes (PAS-positive, magenta), and nuclei (blue).

### 2.9 Measurement of intracellular labile iron (Fe^2+^)

The ferrous iron (Fe^2+^) content was quantified using a Ferrous Iron Colorimetric Assay Kit (Beyotime, S1066S) to assess ferroptosis-relevant iron dysregulation. For RPE/choroid tissue complexes, fresh tissues were homogenized in ice-cold Reagent 1 from the kit, and the supernatant was collected after centrifugation for analysis. For ARPE-19 cells, the cells were washed, lysed directly in Reagent 1, and the lysate was centrifuged to obtain the supernatant. Following the manufacturer’s protocol, 50 µL of each supernatant was reacted with 100 µL of freshly prepared chromogenic working solution in a 96-well plate. After a 30-min incubation at room temperature in the dark, absorbance was measured at 593 nm. A standard curve (0–10 µM Fe^2+^) was used to calculate sample concentrations. Data were normalized to total protein content (determined by BCA assay) and expressed as nmol per mg of protein. Within each independent experiment, the technical replicate values for a given condition were averaged. This average was then normalized to the mean value of the control group, which was set as 1.

### 2.10 Enzyme-linked immunosorbent assay (ELISA)

The concentrations of IL-6 and IL-8 in cell culture supernatants were quantified using commercial ELISA kits (IL-6: Invitrogen, 88-7066; IL-8: Invitrogen, 88-8086) following the manufacturer’s instructions. Supernatants were collected and centrifuged to remove debris. Samples and standards were added to the pre-coated plates and incubated according to the kit specifications. After the final incubation with substrate solution, absorbance was measured at 450 nm with 570 nm reference, and cytokine concentrations were calculated from a standard curve. Each sample was assayed in technical duplicate. The average of the duplicate measurements for each experimental condition within a single experiment was calculated and treated as one biological data point (n = 1). The data are presented as the absolute cytokine concentration in the supernatant (pg/mL).

### 2.11 Measurement of malondialdehyde (MDA)

Lipid peroxidation was assessed by quantifying malondialdehyde (MDA) levels using a commercial Lipid Peroxidation MDA Assay Kit (Beyotime, S0131) based on the thiobarbituric acid (TBA) method. Due to the limited tissue mass of individual RPE/choroid complexes, four complexes from the same experimental group were pooled and processed together as one biological sample for subsequent analysis. Data derived from each such pool were recorded and analyzed as n = 1. Briefly, RPE/choroid complexes were homogenized, and the supernatant was collected after centrifugation. The supernatant was reacted with the TBA working solution at 95 °C for 30–40 min. After cooling, the absorbance of the resulting red product was measured at 532 nm. The MDA concentration for each sample was calculated against a standard curve and normalized to its total protein content (determined by BCA assay). For inter-group comparison, the normalized MDA value of each individual mouse was further normalized to the average value of the 8-week-old mouse group, which was set as 1.

### 2.12 Statistical analysis

Data from at least three independent biological replicates are expressed as the mean ± standard error of the mean (SEM). The sample size *n* denotes the number of independent experiments (conducted with distinct cell passages) or individual animal samples. Prior to parametric testing, the normality of data distribution was assessed using the Shapiro-Wilk test. The homogeneity of variances was verified using Levene’s test (or Brown-Forsythe test where appropriate). Statistical comparisons between two groups were performed using an unpaired two-tailed Student’s t-test. Comparisons among multiple groups were analyzed by one-way analysis of variance (ANOVA) followed by Tukey’s *post hoc* test. Statistical analysis was conducted using GraphPad Prism 9.0 software. A *p*-value of <0.05 was considered statistically significant (*P < 0.05, **P < 0.01, ***P < 0.001, ***P < 0.0001).

## 3 Results

### 3.1 Age-related changes in the murine RPE/choroid complex

**FIGURE 1:**
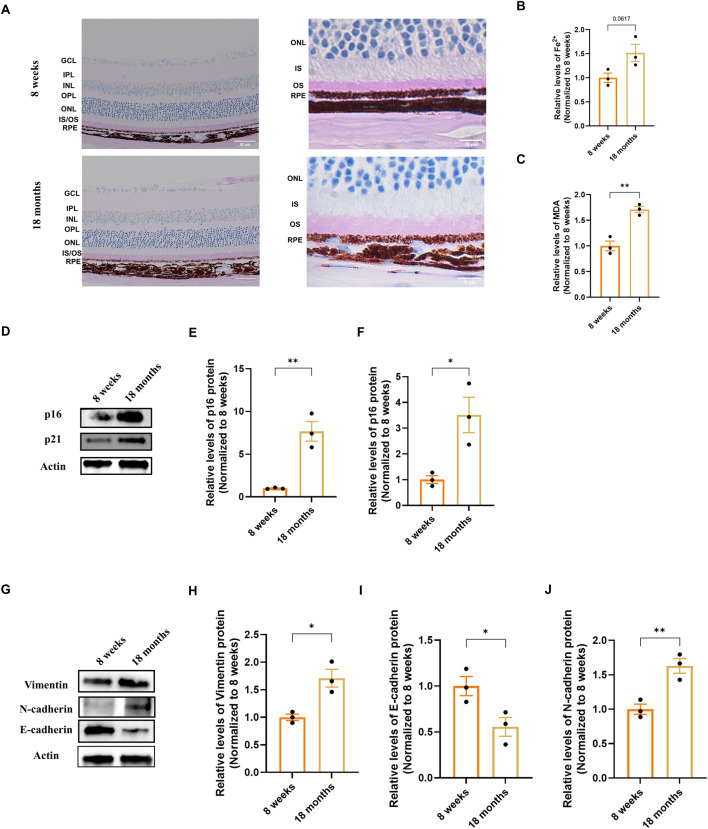
Characterization of senescence, ferroptosis, and EMT markers in the RPE/choroid complex of naturally aging mice. **(A)** Representative Periodic Acid–Schiff (PAS) stained retinal sections from 8-week-old and 18-month-old mice, imaged at 20× (scale bar: 50 µm) and 100× (scale bar: 20 µm) magnification. Images were captured at a standard location approximately 500 μm from the optic nerve head. **(B,C)** Quantification of ferroptosis-related markers in RPE/choroid complexes (n = 3). **(B)** Ferrous iron (Fe^2+^) content. **(C)** Malondialdehyde (MDA) levels, indicative of lipid peroxidation. Data are normalized to total protein and presented relative to the young group (set as 1). **(D–F)** Analysis of senescence markers. **(D)** Representative Western blot images for p16 and p21. **(E,F)** Densitometric quantification of p16 and p21 protein levels (n = 3). **(G)** Representative Western blot images for Vimentin, N-cadherin and E-cadherin. **(H-J)** Densitometric quantification of Vimentin, N-cadherin and E-cadherin protein levels (n=3). Band intensities were first normalized to β-actin and then calibrated to the mean value of the young control group (set as 1). Data are presented as mean ± SEM (n = 3 biologically independent samples per group). Statistical significance was determined by unpaired two-tailed Student’s t-test (*p < 0.05, **p < 0.01, ***p < 0.001).

To investigate early pathogenic changes relevant to age-related macular degeneration (AMD), we first examined key ferroptosis and epithelial-mesenchymal transition (EMT) markers in naturally aging mice. Periodic acid–Schiff (PAS) staining showed a well-preserved laminar retinal structure in 18-month-old mice; however, thickening of the outer segment (OS) layer was observed, which is presumed to be associated with a decline in RPE phagocytic function (Figure 1A). We next analyzed hallmarks of ferroptosis. We found an accumulation of ferrous iron (Fe^2+^) in the RPE/choroid complexes of aged mice (Figure 1B), a finding consistent with clinical observations of iron dysregulation in AMD (Hahn et al., 2003; Biesemeier et al., 2015). Concurrently, the level of malondialdehyde (MDA), a marker of lipid peroxidation, was also elevated (Figure 1C).

We then evaluated markers of cellular senescence. Western blot analysis showed a significant upregulation of the cyclin-dependent kinase inhibitors p16 and p21 in the aged tissue compared to young controls (Figures 1D–F). Finally, we analyzed the expression of classic EMT markers. Protein analysis demonstrated a significant increase in the mesenchymal markers Vimentin and N-cadherin, alongside a decrease in the epithelial adhesion protein E-cadherin in 18-month-old mice (Figures 1G–J). Together, these results indicate that the RPE/choroid complex of natural aging mice is accompanied by iron accumulation, lipid peroxidation, senescence marker upregulation, and a shift in the expression of EMT-associated proteins.

### 3.2 Establishment of D-galactose-induced senescence and sodium iodate-induced injury models *in vitro*

**FIGURE 2:**
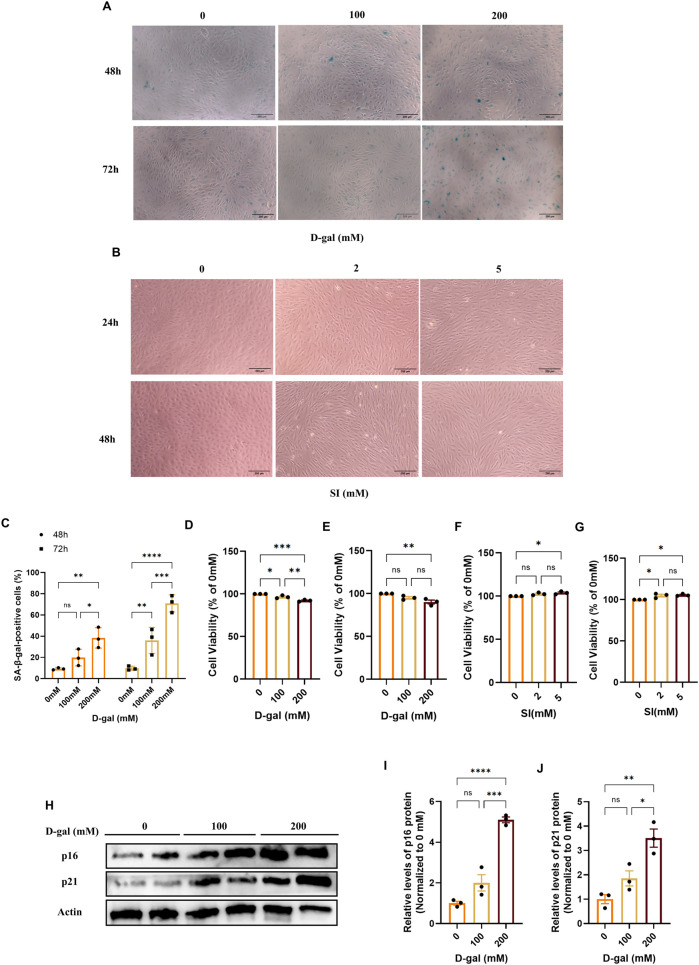
Establishment and characterization of D-galactose and sodium iodate-induced models in ARPE-19 cells. **(A)** Representative images of senescence-associated β-galactosidase (SA-β-gal) staining in ARPE-19 cells treated with D-gal at the indicated concentrations and time points (with a 10× objective, scale bar: 200 µm). **(B)** Phase-contrast images showing the morphological changes in ARPE-19 cells treated with SI at the indicated concentrations and time points (with a 10× objective, scale bar: 200 µm). **(C)** Quantitative analysis of SA-β-gal-positive cells from **(A)**. Data are presented as the percentage of positive cells (n = 3). **(D,E)** Cell viability assessed by CCK-8 assay after treatment with D-gal for 48 **(D)** or 72 **(E)** hours (n = 3). **(F,G)** Cell viability assessed by CCK-8 assay after treatment with SI for 24 **(F)** or 48 **(G)** hours (n = 3). **(H)** Representative Western blot images showing the protein levels of the senescence markers p16 and p21 in cells treated with increasing concentrations of D-gal for 72 h. **(I,J)** Densitometric quantification of p16 **(I)** and p21 **(J)** protein levels from **(H)** (n = 3). Band intensities were normalized to β-actin and are presented relative to the control group (0 mM, PBS, mean set as 1). Data are presented as mean ± SEM from at least three independent experiments. Statistical significance was determined by one-way ANOVA with Tukey’s *post hoc* test (p < 0.05, **p < 0.01, **p < 0.001).

To dissect stage-specific mechanisms of RPE pathology in AMD, we established complementary *in vitro* models. We employed D-galactose (D-gal) to model the chronic, progressive stress associated with early-to-intermediate AMD, which primarily triggers a senescence-like state (Li et al., 2023; Ida et al., 2004; Azman and Zakaria, 2019). Treatment with D-gal induced a dose- and time-dependent increase in SA-β-galactosidase activity (Figures 2A,C) and upregulated senescence markers p16 and p21 (Figures 2H–J). The condition of 200 mM D-gal for 72 h was selected for subsequent studies, as it induced a robust senescence phenotype (SA-β-gal-positive cells >60%) while maintaining viability above 80% (Figures 2C–E).

To model the acute, intense oxidative injury relevant to late-stage AMD pathology, we utilized sodium iodate (SI), which has been increasingly recognized as a relevant *in vitro* system for retinal degeneration (Enzbrenner et al., 2021; Anderson et al., 2024). SI treatment induced distinct morphological alterations, including cell elongation and loss of epithelial cobblestone structure (Figure 2B). The condition of 2 mM SI for 48 h was chosen, as it produced these characteristic changes under conditions of preserved viability (Figures 2B,F,G), enabling the study of injury-driven phenotypic transition.

Collectively, these data define two distinct cellular models, a D-gal-induced senescence model and an SI-induced oxidative injury model, that provide sub-cytotoxic platforms to investigate complementary aspects of AMD pathogenesis.

### 3.3 D-galactose and sodium iodate induce epithelial-mesenchymal transition in ARPE-19 cells

**FIGURE 3:**
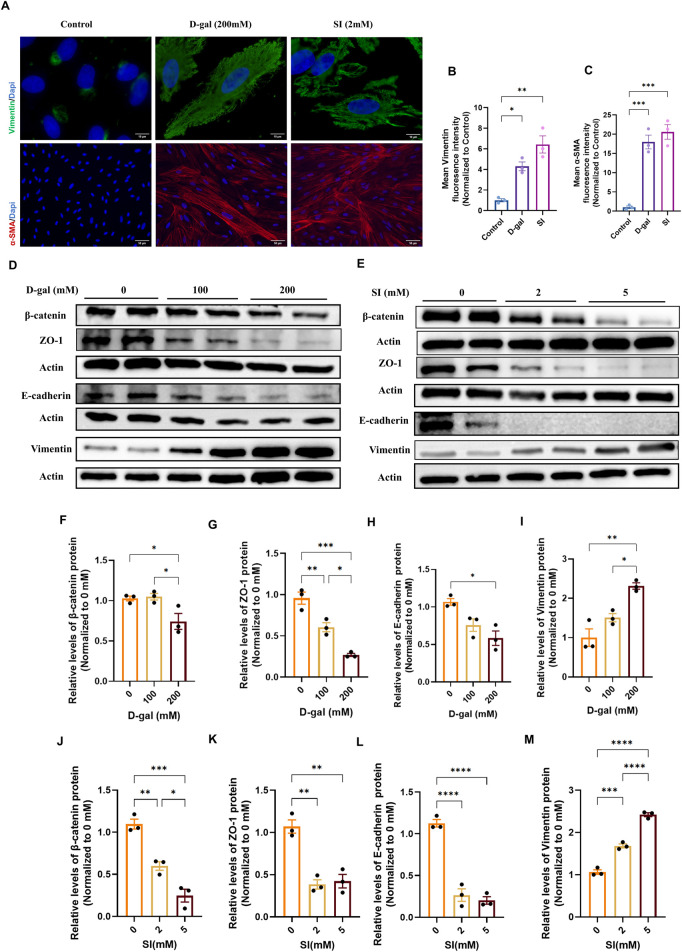
D-galactose and sodium iodate induce epithelial-mesenchymal transition in ARPE-19 cells. **(A)** Representative immunofluorescence images of differentiated ARPE-19 cells stained for the mesenchymal markers Vimentin (green) and α-smooth muscle actin (α-SMA, red). Images show cells under control conditions, or following treatment with D-gal (200 mM, 72 h; acquired with a 100× objective, scale bar: 10 µm) or SI (2 mM, 48 h; acquired with a ×20 objective, scale bar: 50 µm). **(B,C)** Quantitative analysis of the mean fluorescence intensity (MFI) for Vimentin **(B)** and α-SMA **(C)**. Fluorescence intensity was normalized to the control group (set as 1) (n = 3). **(D)** Treatment with 100 mM and 200 mM D-gal significantly reduced the expression of epithelial/junctional proteins β-catenin **(F)**, ZO-1 **(G)**, and E-cadherin **(H)**, while upregulating the mesenchymal marker Vimentin **(I)** in a dose-dependent manner (n = 3). **(E)** Treatment with 2 mM and 5 mM SI promoted similar alterations in EMT markers, reducing β-catenin **(J)**, ZO-1 **(K)**, and E-cadherin **(L)**, and increasing Vimentin **(M)** expression (n = 3). Densitometric quantification is normalized to β-actin and presented relative to the control (0 mM, PBS, set as 1). Data are presented as mean ± SEM from at least three independent experiments. Statistical significance was determined by one-way ANOVA with Tukey’s *post hoc* test (p < 0.05, **p < 0.01, ***p < 0.001, ***p < 0.0001).

We next investigated whether the pathological stimuli modeled by D-gal and SI could drive the loss of epithelial phenotype. Immunofluorescence analysis revealed that both treatments markedly increased the expression and induced cytoskeletal reorganization of the mesenchymal markers Vimentin and α-smooth muscle actin (α-SMA) compared to controls (Figure 3A). Quantitative analysis confirmed increases in the mean fluorescence intensity for both markers, with the induction being more pronounced in SI-treated cells (Figures 3B,C).

To further characterize this phenotypic shift at the molecular level, we analyzed a panel of epithelial and mesenchymal markers by Western blot. Consistent with the immunofluorescence results, D-gal treatment caused a dose-dependent downregulation of the epithelial/junction proteins ZO-1, β-catenin, and E-cadherin, alongside an upregulation of Vimentin (Figures 3D,F–I). Similarly, SI treatment induced a more drastic loss of epithelial markers and a robust gain of Vimentin expression (Figures 3E,J–M). These coordinated changes in protein expression profile suggest that both chronic senescence-inducing stress (D-gal) and acute oxidative injury (SI) are potent inducers of EMT in RPE cells.

### 3.4 D-galactose and sodium iodate trigger ferroptotic stress in ARPE-19 cells

**FIGURE 4:**
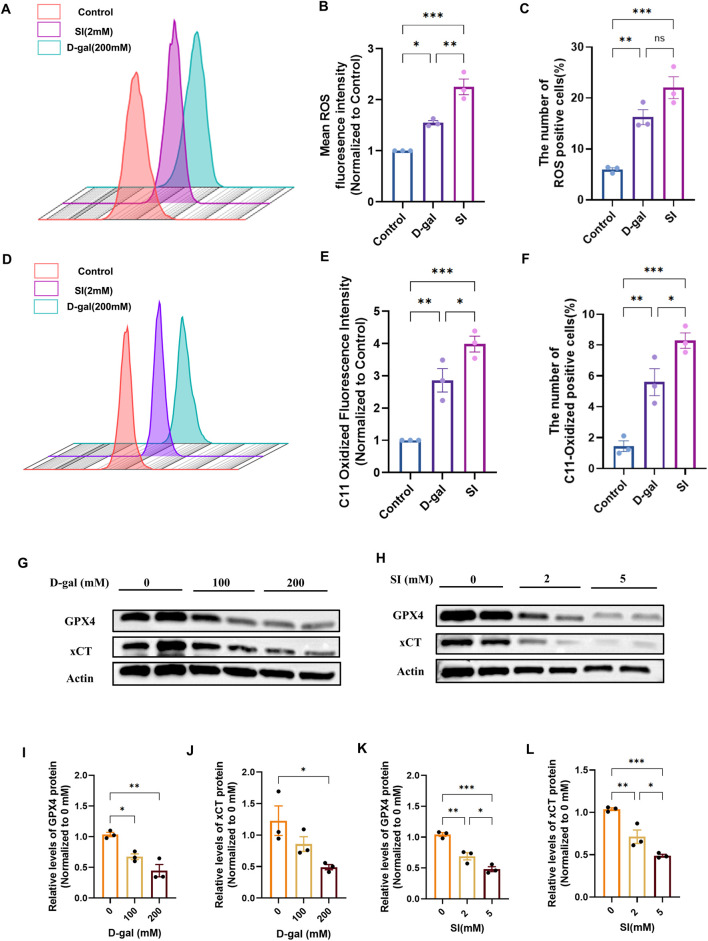
D-galactose and sodium iodate trigger ferroptotic stress in ARPE-19 cells. **(A)** Flow cytometric analysis of intracellular reactive oxygen species (ROS) levels using the DCFH-DA probe in cells treated with D-gal or SI. **(B,C)** Quantitative analysis of the geometric mean fluorescence intensity (MFI) **(B)** and the percentage of ROS-positive cells **(C)** from **(A)** (n = 3). **(D)** Flow cytometric analysis of lipid peroxidation using the C11-BODIPY 581/591 probe in cells treated with D-gal or SI. **(E,F)** Quantitative analysis of the geometric MFI **(E)** and the percentage of C11-BODIPY-positive cells **(F)** from **(D)** (n = 3). **(G,H)** Representative Western blot images showing protein levels of key ferroptosis regulators, xCT and GPX4, in cells treated with increasing concentrations of D-gal **(G)** or SI **(H)**. **(I,J)** Densitometric quantification of xCT **(I)** and GPX4 **(J)** protein levels from **(G)**. Data are normalized to β-actin and presented relative to the untreated control (set as 1). **(K,L)** Densitometric quantification of xCT **(K)** and GPX4 **(L)** protein levels from **(H)** (n = 3). Data are normalized to β-actin and presented relative to the untreated control (set as 1). Data are presented as mean ± SEM from three independent experiments. Statistical significance was determined by one-way ANOVA with Tukey’s *post hoc* test (p < 0.05, **p < 0.01, ***p < 0.001, ***p < 0.0001).

Having established that both models induce EMT, we sought to determine if this phenotypic shift was associated with the activation of ferroptosis. We first assessed general oxidative stress and specific lipid peroxidation. Flow cytometric analysis using DCFH-DA revealed a significant increase in intracellular reactive oxygen species (ROS) in cells treated with either D-gal or SI (Figures 4A–C). More specifically, flow cytometric analysis with the lipid peroxidation sensor C11-BODIPY demonstrated an accumulation of oxidized lipids, a hallmark of ferroptosis, in both models (Figures 4D–F). We then examined the expression of core ferroptosis defense proteins (Ding et al., 2024; Ye et al., 2025; Feng et al., 2025). Western blot analysis showed that both D-gal and SI treatment caused a dose-dependent downregulation of xCT and glutathione peroxidase 4 (GPX4) (Figures 4G–L). The coordinated impairment of this key antioxidant axis (xCT/GPX4), alongside the accumulation of lipid peroxides, suggests that both senescence-inducing and acute oxidative insults converge on activating ferroptotic stress pathways in RPE cells.

### 3.5 Ferroptosis is involved in both senescence and oxidative injury-induced EMT

**FIGURE 5:**
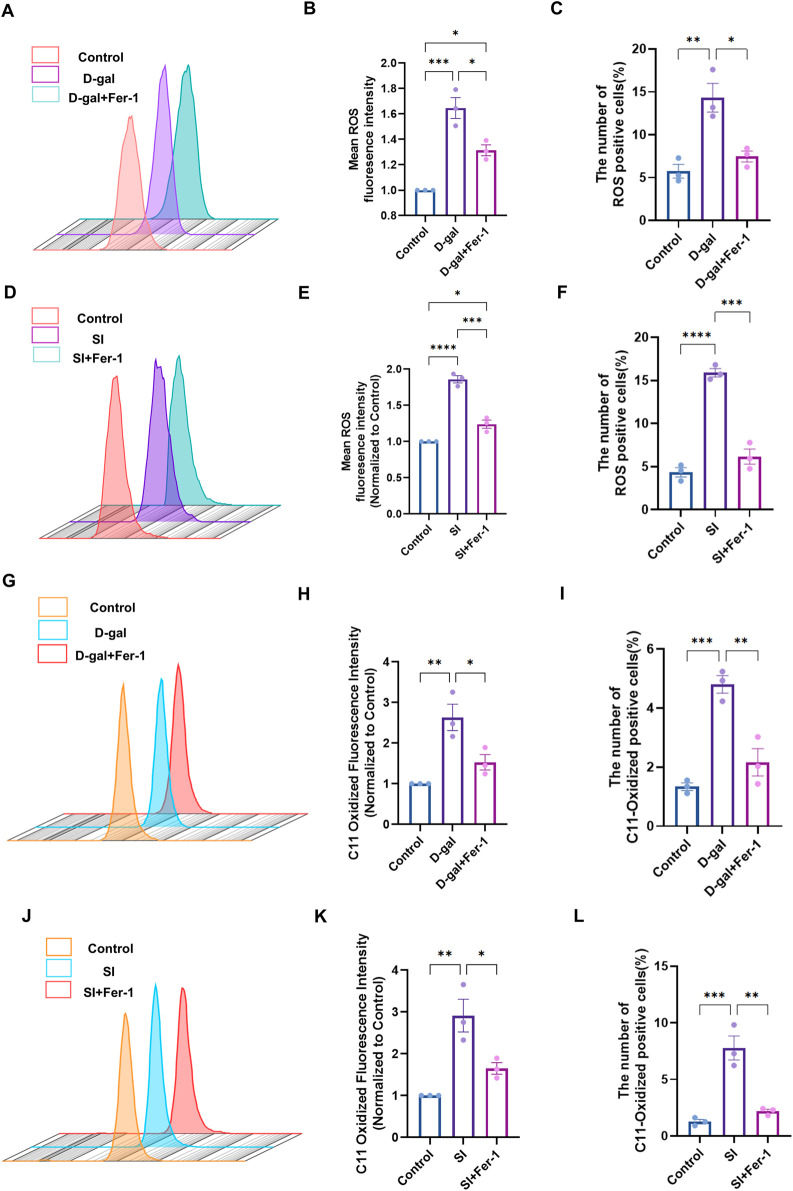
Ferrostatin-1 rescues D-galactose- and sodium iodate-induced oxidative stress and lipid peroxidation. **(A)** Flow cytometric analysis of intracellular reactive oxygen species (ROS) using the DCFH-DA probe in cells treated with D-gal (200 mM, 72 h) with or without Fer-1 (20 µM) pretreatment. **(B,C)** Quantitative analysis of the geometric mean fluorescence intensity (MFI) **(B)** and the percentage of ROS-positive cells **(C)** from **(A)** (n = 3). **(D)** Flow cytometric analysis of ROS in cells treated with SI (2 mM, 48 h) with or without Fer-1 pretreatment. **(E,F)** Quantitative analysis of the MFI **(E)** and the percentage of ROS-positive cells **(F)** from **(D)** (n = 3). **(G)** Flow cytometric analysis of lipid peroxidation using the C11-BODIPY 581/591 probe in D-gal-treated cells with or without Fer-1. **(H,I)** Quantitative analysis of the MFI **(H)** and the percentage of C11-BODIPY-positive cells **(I)** from **(G)** (n = 3). **(J)** Flow cytometric analysis of lipid peroxidation in SI-treated cells with or without Fer-1. **(K,L)** Quantitative analysis of the MFI **(K)** and the percentage of C11-BODIPY-positive cells **(L)** from **(J)**. Data are presented as mean ± SEM from three independent experiments. Statistical significance was determined by one-way ANOVA with Tukey’s *post hoc* test (p < 0.05, **p < 0.01, **p < 0.001).

**FIGURE 6:**
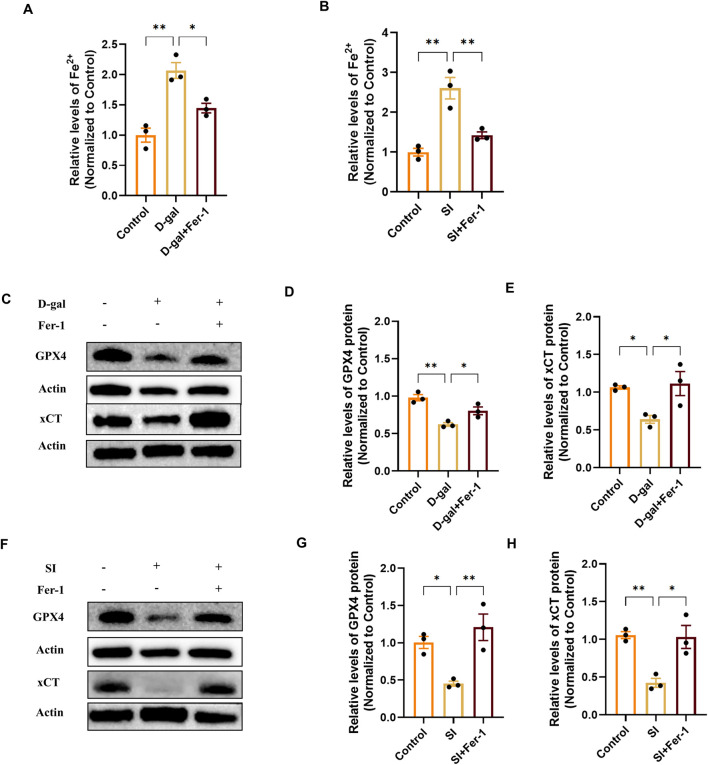
Ferrostatin-1 mitigates ferroptosis by reducing iron overload and restoring the xCT/GPX4 defense axis. **(A,B)** Quantification of intracellular ferrous iron (Fe^2+^) content showing that Fer-1 (20 µM) pretreatment attenuates the increase induced by D-gal **(A)** or SI **(B)**. Data are normalized to total protein and presented relative to the control group (set as 1) (n = 3). **(C)** Representative Western blot images showing that Fer-1 restores the protein levels of xCT and GPX4 downregulated by D-gal treatment. **(D,E)** Densitometric quantification of xCT **(D)** and GPX4 **(E)** protein levels from **(C)** (n = 3). **(F)** Representative Western blot images showing that Fer-1 restores the protein levels of xCT and GPX4 downregulated by SI treatment. **(G,H)** Densitometric quantification of xCT **(G)** and GPX4 **(H)** protein levels from **(F)** (n = 3). Data are presented as mean ± SEM from three independent experiments. Statistical significance was determined by one-way ANOVA with Tukey’s *post hoc* test (p < 0.05, **p < 0.01, **p < 0.001).

Having established that ferroptosis hallmarks are activated in both the D-gal and SI models (Figure 4), we next sought to determine whether this pathway was functionally required for the observed pathology. We therefore employed the specific ferroptosis inhibitor Ferrostatin-1 (Fer-1). Fer-1 pretreatment effectively mitigated the downstream execution events. It significantly attenuated the accumulation of intracellular reactive oxygen species (ROS) and the oxidation of the lipid peroxidation sensor C11-BODIPY induced by both D-gal and SI (Figure 5). Furthermore, Fer-1 targeted key upstream drivers of the ferroptotic cascade. It reduced the pathological increase in the labile iron pool (Fe^2+^) (Figures 6A,B) and concurrently restored the protein levels of the core anti-ferroptotic components xCT and GPX4, which were downregulated by both stimuli (Figures 6C–H). The coordinated rescue of these hallmark events suggests that ferroptosis might be functionally involved in the pathological cascade triggered by both chronic senescence-like stress (D-gal) and acute oxidative injury (SI) in RPE cells.

### 3.6 Inhibition of ferroptosis attenuates D-galactose-induced senescence

**FIGURE 7:**
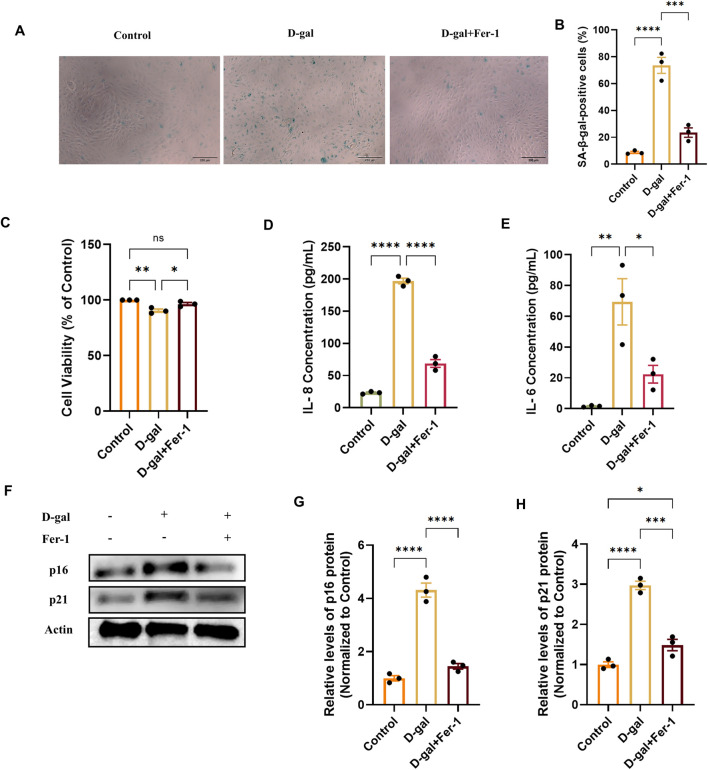
Ferrostatin-1 alleviates D-galactose-induced cellular senescence in ARPE-19 cells. **(A)** Representative images of SA-β-galactosidase (SA-β-gal) staining in cells treated with D-gal (200 mM, 72 h) with or without pretreatment with the ferroptosis inhibitor Ferrostatin-1 (Fer-1, 20 µM) (acquired with a 10× objective, scale bar: 200 µm). **(B)** Quantification of SA-β-gal-positive cells from **(A)** (n = 3). **(C)** Cell viability assessed by CCK-8 assay in the indicated treatment groups (n = 3). **(D,E)** Concentrations of the SASP factors IL-8 **(D)** and IL-6 **(E)** in cell culture supernatants, measured by ELISA (n = 3). **(F)** Representative Western blot images showing protein levels of the senescence markers p21 and p16. **(G,H)** Densitometric quantification of p21 **(G)** and p16 **(H)** protein levels from **(F)**. Band intensities were normalized to β-actin and are presented relative to the control group (set as 1) (n = 3). Data are presented as mean ± SEM from three independent experiments. Statistical significance was determined by one-way ANOVA with Tukey’s *post hoc* test (p < 0.05, **p < 0.01, **p < 0.001).

To investigate whether ferroptosis is a causative mechanism involved in the observed senescence, we inhibited ferroptosis using Ferrostatin-1 (Fer-1) in the D-gal-induced model. Pretreatment with 20 µM Fer-1 for 2 h reduced the proportion of SA-β-gal-positive cells induced by D-gal (Figures 7A,B) and restored cell viability (Figure 7C). Furthermore, Fer-1 treatment decreased the secretion of the senescence-associated secretory phenotype (SASP) factors IL-8 and IL-6 (Figures 7D,E). At the molecular level, the upregulation of the key senescence effector proteins p21 and p16 by D-gal was effectively suppressed by Fer-1 (Figures 7F–H). These results demonstrate that ferroptosis inhibition may attenuate multiple hallmarks of cellular senescence, positioning ferroptosis as a potentially upstream regulatory event in D-gal-induced RPE senescence.

### 3.7 Inhibition of ferroptosis rescues EMT phenotypes in both senescence and oxidative injury models

**FIGURE 8:**
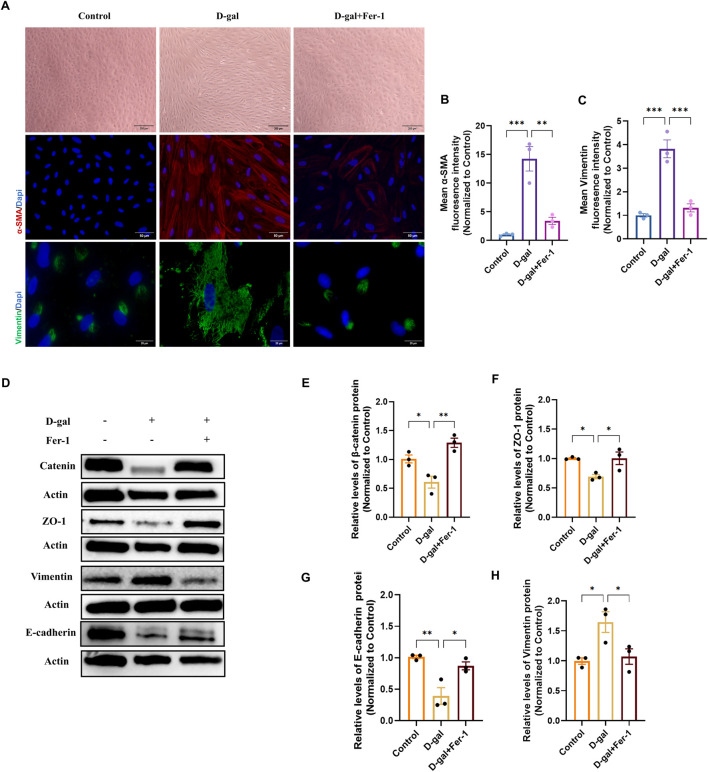
Ferrostatin-1 rescues D-galactose-induced epithelial-mesenchymal transition in ARPE-19 cells. **(A)** Phase-contrast (10×, scale bar: 200 µm) and representative immunofluorescence images of α-SMA (20×, scale bar: 50 µm) and Vimentin (100×, scale bar: 20 µm) in control, D-gal-treated, and D-gal + Fer-1-treated cells. **(B,C)** Quantitative analysis of the mean fluorescence intensity (MFI) for α-SMA **(B)** and Vimentin **(C)** from **(A)** (n = 3). **(D)** Representative Western blot images showing protein levels of the epithelial/junction markers β-catenin, ZO-1, and E-cadherin, and the mesenchymal marker Vimentin. **(E–H)** Densitometric quantification of β-catenin **(E)**, ZO-1 **(F)**, E-cadherin **(G)**, and Vimentin **(H)** protein levels from **(D)** (n = 3). Data in all panels are presented as mean ± SEM from three independent experiments. Statistical significance was determined by one-way ANOVA with Tukey’s *post hoc* test (p < 0.05, **p < 0.01, **p < 0.001).

To determine whether inhibiting ferroptosis could reverse the established EMT phenotype, we treated cells with Ferrostatin-1 (Fer-1) in both the D-gal and SI models. In the D-gal-induced senescence model, Fer-1 pretreatment partially restored the epithelial cobblestone morphology (Figure 8A). Concurrently, it reduced the expression of the mesenchymal markers α-SMA and Vimentin, as quantified by mean fluorescence intensity (Figures 8A–C). At the molecular level, Western blot analysis confirmed that Fer-1 rescued the D-gal-induced downregulation of the epithelial/junction proteins β-catenin, ZO-1, and E-cadherin, while suppressing the upregulation of Vimentin (Figures 8D–H).

**FIGURE 9:**
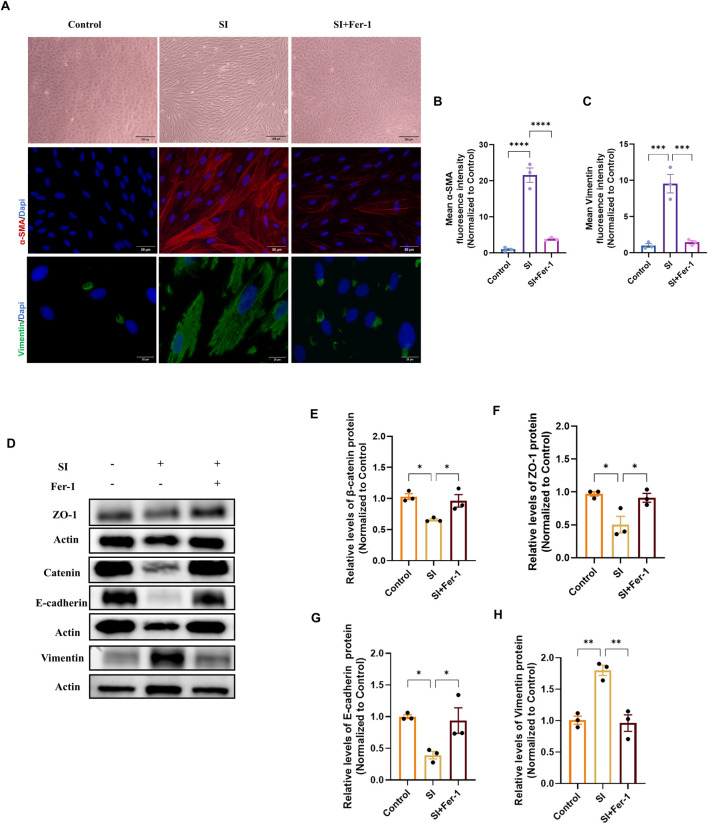
Ferrostatin-1 rescues sodium iodate-induced epithelial-mesenchymal transition in ARPE-19 cells. **(A)** Phase-contrast (10×, scale bar: 200 µm) and representative immunofluorescence images of α-SMA (20×, scale bar: 50 µm) and Vimentin (100×, scale bar: 20 µm) in control, SI-treated, and SI + Fer-1-treated cells. **(B,C)** Quantitative analysis of the mean fluorescence intensity (MFI) for α-SMA **(B)** and Vimentin **(C)** from **(A)** (n = 3). **(D)** Representative Western blot images showing protein levels of β-catenin, ZO-1, E-cadherin, and Vimentin. **(E–H)** Densitometric quantification of β-catenin **(E)**, ZO-1 **(F)**, E-cadherin **(G)**, and Vimentin **(H)** protein levels from **(D)** (n = 3). Data in all panels are presented as mean ± SEM from three independent experiments. Statistical significance was determined by one-way ANOVA with Tukey’s *post hoc* test (p < 0.05, **p < 0.01, **p < 0.001).

An identical protective pattern was observed in the SI-induced oxidative injury model. Fer-1 ameliorated the elongated, mesenchymal-like morphology and attenuated the induction of α-SMA and Vimentin (Figures 9A–C). Similarly, it restored the expression of epithelial markers and decreased Vimentin at the protein level (Figures 8D–H).

These parallel rescue effects across two distinct models demonstrate that inhibiting ferroptosis can restore epithelial homeostasis, highlighting its potential as a therapeutic target for pathological RPE.

## 4 Discussion

In this study, we sought to investigate the role of ferroptosis in age-related alterations of the retinal pigment epithelium by employing three complementary models designed to capture different facets of AMD pathology: natural aging (early metabolic shifts), D-gal-induced senescence (chronic oxidative stress, relevant to intermediate stages), and low-dose SI-induced injury (acute oxidative stress/EMT, modeling late-stage complications such as fibrosis). Across these models, we observed a consistent upregulation of ferroptosis hallmarks and EMT markers, albeit with varying degrees of intensity across different contexts. Notably, the ferroptosis inhibitor Ferrostatin-1 (Fer-1) was able to mitigate these changes in both the D-gal and SI cellular models, suggesting a functional link between ferroptosis, senescence, and EMT in the RPE.

Prior study from Sun et al. has reported senescence, EMT, and ferroptosis as co-occurring pathological phenomena in RPE (Sun et al., 2018). Our work builds upon this foundation and specifically investigates the functional interplay between these pathways. The role of ferroptosis in AMD pathogenesis has been supported by evidence from various AMD models (Tang et al., 2021; Huang et al., 2025) and observations that iron overload or ferroptosis inducers can recapitulate AMD-like pathology (Liu et al., 2021; Tong and Wang, 2020). In this context, while high-dose SI is a well-established model for inducing RPE degeneration linked to ferroptosis (Henning et al., 2022; Upadhyay and Bonilha, 2024; Xiang et al., 2024), recent work by Yang et al. (2024) has proposed a relationship between low-dose SI and EMT changes in RPE cells. Our findings are consistent with this conclusion, and we have further tested and demonstrated the rescuing effect of the ferroptosis inhibitor Ferrostatin-1 in this specific model.

More importantly, we have expanded this association to the D-gal-induced senescence and natural aging models. The connection between ferroptosis and retinal aging has been suggested, with studies reporting increased ferroptosis markers in retinal ganglion cells with age (Wang et al., 2025b; Zhao et al., 2021). However, directly linking RPE ferroptosis to cellular senescence remains relatively underexplored. A pivotal study linked iron overload to TBH (tert-butyl hydroperoxide) -induced senescence in ARPE-19 cells and D-gal-induced aging in mice (Li et al., 2023). Our study, utilizing three distinct models, similarly confirms the important role of ferroptosis in RPE senescence and extends these findings by establishing its connection to the downstream EMT process.

Changes in EMT-related markers have also been detected in AMD samples (Gurubaran et al., 2025) and it has been reported that clearing senescent cells or administering anti-aging treatments can inhibit EMT alterations in the RPE (Jang et al., 2023; Jiang et al., 2025; Gao et al., 2022). Our data from natural aging and D-gal models provide mechanistic support for these observations. Taken together, our findings move beyond describing coexistence and suggest an integrated pathogenic axis. This leads us to propose that senescence may trigger EMT changes, with ferroptosis serving as a potential linking mechanism.

Several limitations of this study must be acknowledged, as they define important future directions. First, the use of only male mice limits the generalizability of our findings. Second, while D-gal and SI are valuable chemical inducers, complementary genetic models would strengthen the causal evidence, and direct investigation of ferroptosis inhibitors in aging mouse models is crucial to validate the translational relevance of our findings *in vivo*. Third, our study does not fully delineate the molecular intermediates linking ferroptosis to the EMT transcriptional program. Finally, while our interventional data using Fer-1 strongly support the necessity of ferroptosis in the observed pathology, future studies employing direct ferroptosis inducers (gain-of-function approaches) would help to further establish its sufficiency in driving senescence and EMT. Additionally, investigating these mechanisms in combined “multiple-hit” models could better simulate the complexity of advanced disease. Our findings should be interpreted with caution due to the limitations mentioned above.

In conclusion, this study represents a systematic attempt to dissect the connections between aging, ferroptosis, and EMT in the RPE. We propose that ferroptosis may play a significant role in influencing senescence and thereby affecting EMT progression. Building on these findings, future research should focus on whether endogenous protective factors like Klotho confer resilience by modulating ferroptosis, and on validating the therapeutic potential of ferroptosis inhibition in more complex *in vivo* models.

## Data Availability

The original contributions presented in the study are included in the article/supplementary material, further inquiries can be directed to the corresponding authors.
